# Imprint of parity and age at first pregnancy on the genomic landscape of subsequent breast cancer

**DOI:** 10.1186/s13058-019-1111-6

**Published:** 2019-02-15

**Authors:** Bastien Nguyen, David Venet, Matteo Lambertini, Christine Desmedt, Roberto Salgado, Hugo Mark Horlings, Françoise Rothé, Christos Sotiriou

**Affiliations:** 1 Breast Cancer Translational Research Laboratory J.-C. Heuson, Institut Jules Bordet, Université Libre de Bruxelles (ULB), Brussels, Belgium; 2 Present Address: Marie-Josée and Henry R. Kravis Center for Molecular Oncology, Memorial Sloan Kettering Cancer Center, New York, USA; 3 Department of Pathology, GZA-ZNA, Antwerp, Belgium; 4 Department of Pathology, The Netherlands Cancer Institute, Amsterdam, The Netherlands; 5 Department of Medical Oncology, Clinica di Oncologia Medica, Ospedale Policlinico San Martino, & Department of Internal Medicine and Medical Specialties (DiMI), School of Medicine, University of Genova, Genova, Italy

**Keywords:** Breast cancer, Pregnancy, Genomics, SCNAs, Mutational landscape, Whole genome sequencing, PABC

## Abstract

**Background:**

Although parity and age at first pregnancy are among the most known extrinsic factors that modulate breast cancer risk, their impact on the biology of subsequent breast cancer has never been explored in depth. Recent data suggest that pregnancy-induced tumor protection is different according to breast cancer subtypes, with parity and young age at first pregnancy being associated with a marked reduction in the risk of developing luminal subtype but not triple negative breast cancer. In this study, we investigated the imprint of parity and age at first pregnancy on the pattern of somatic mutations, somatic copy number alterations, transcriptomic profiles, and tumor immune microenvironment by assessing tumor-infiltrating lymphocytes (TILs) levels of subsequent breast cancer.

**Methods:**

A total of 313 patients with primary breast cancer with available whole genome, RNA sequencing, and TILs data were included in this study. We used a multivariate analysis adjusted for age at diagnosis, pathological stage, molecular subtypes, and histological subtypes. We compared nulliparous vs. parous, late parous vs. early parous, and nulliparous vs. pregnancy-associated breast cancer (PABC) patients. Late and early parous patients were grouped by using the median age at first pregnancy. PABC was defined as patients diagnosed up to 10 years postpartum.

**Results:**

Genomic alterations of breast cancer were associated with age at first pregnancy but not with parity status alone. Independently of clinicopathological features, early parous patients developed tumors characterized by a higher number of Indels (*P*_adj_ = 0.002), a lower frequency of *CDH1* mutations (1.2% vs. 12.7%; *P*_adj_ = 0.013), a higher frequency of *TP53* mutations (50% vs. 22.5%; *P*_adj_ = 0.010), and *MYC* amplification (28% vs. 7%; *P*_adj_ = 0.008). PABC were associated with increased TILs infiltration (*P*_adj_ = 0.0495).

**Conclusions:**

These findings highlight an unprecedented link between reproductive history and the genomic landscape of subsequent breast cancer. We further hypothesize that *TP53*-mutant premalignant lesions could be less susceptible to the protective effect of an early parity, which might explain the difference of parity-induced protection according to breast cancer subtypes. This work also advocates that reproductive history should be routinely collected in future large-scale genomic studies addressing the biology of female cancers.

**Electronic supplementary material:**

The online version of this article (10.1186/s13058-019-1111-6) contains supplementary material, which is available to authorized users.

## Background

The effect of parity and age at first pregnancy on the risk of developing breast cancer has been well documented [1–5]. Parity is known to have a dual effect on breast cancer risk with an increased risk during 5 to 10 years after pregnancy, followed by a strong and life-long protective effect [1, 6]. This effect is strongly influenced by age at first pregnancy as pregnancy-induced tumor protection is more pronounced if first pregnancy has occurred early in life. Recent data suggest that pregnancy-induced tumor protection is different according to breast cancer subtypes, with parity and young age at first pregnancy being associated with a marked reduction in the risk of developing luminal subtype tumors [7–10].

Several studies have attempted to investigate the mechanisms underlying this phenomenon [11, 12]. However, although parity and age at first pregnancy are among the most known extrinsic factors that modulate breast cancer risk, their impact on the biology of breast cancer has never been explored in depth. We have previously observed that breast cancer diagnosed during pregnancy has different biology at the genomic levels [13]. In the present study, we used a systematic multivariate analysis to investigate the imprint of parity and age at first pregnancy on the pattern of somatic mutations, somatic copy number alterations (SCNAs), transcriptomic profiles, and tumor-infiltrating lymphocytes (TILs) levels in a series of 313 breast cancer patients with available whole genome, RNA sequencing, and TILs levels data.

## Methods

### Data acquisition

All analyses were performed on a publicly available dataset comprising 560 breast cancer patients referred to as BRCA560 [14]. Clinical data, sequencing coverage, and mutational load were obtained from Additional file 1: Table S1–S3 in that reference. Coding driver mutation events and the contribution of mutational signatures were obtained from Additional file 1: Table S14 and S21 in that reference. Raw count data from RNA sequencing were obtained from the authors. Results from HRDetect classifier were obtained from Additional file 1: Table S4 in reference [15].

### Patients selection

Eligible patients from BRCA560 were those with samples collected from primary tumor only (patients with local recurrence or metastasis samples were excluded, *n* = 8) who had available information on parity. There were only two available HER2+ patients (both parous) in the transcriptomic analysis so we preferred to exclude them from this analysis. For each patient, we determined the breast cancer intrinsic subtype (PAM50) using genefu R/Bioconductor package [16]. Nulliparous patients were defined as women with breast cancer who had no full-term pregnancy at the time of breast cancer diagnosis. Parous patients were defined as women with breast cancer who had at least one full-term pregnancy at the time of breast cancer diagnosis. Early parous patients were defined as ≤ 25 years of age at first full-term pregnancy, while late parous patients were defined as > 25 years of age at first full-term pregnancy. PABC patients were defined as women diagnosed not during pregnancy but up to 10 years after the first pregnancy. As referred to the publicly available BRCA560 dataset [14], the Internal Review Boards of each participating institution approved the collection and use of samples of all patients in this study.

### TIL evaluation

The percentage of TILs was independently evaluated by two pathologists (R.S. and H.M.H.) on hematoxylin and eosin slides using the International TILs Working Group 2014 methodology as described before [17]. There were 242 original samples with evaluable TILs from 239 patients. For the three patients with two samples, the arithmetic averages were obtained. We obtain a final set of 231 patients with primary tumor only (patients with local recurrence or metastasis samples only were excluded, *n* = 8). TIL information for patients for which evaluation from only one pathologist was available were discarded (*n* = 3). The TILs were scored in the context of an International Cancer Genome Consortium (ICGC) project on the immune characterization of this series, and the scoring is available for all institutions having the ICGC Data Access Compliance Office (DACO) approval. The data will be made broadly available when this global project will be finished or before upon request to the authors.

### Statistical analysis

Except for age at diagnosis that was considered as a continuous variable and therefore compared using the non-parametric Mann–Whitney *U* test, differences in other clinicopathological characteristics of breast cancer between groups were analyzed using the *χ*^2^ test or the Fisher exact test when appropriate. All statistical tests comparing groups were done using the non-parametric Mann–Whitney *U* test and the χ2 test or the Fisher exact test when appropriate for continuous and categorical variables, respectively. For the multivariate analysis, we used a linear and logistic regression to assess the independent association of continuous (log transformed) and categorical variables respectively with—parity (nulliparous vs parous) or—age at first pregnancy (≤ 25 years vs. > 25 years) controlling for age at diagnosis, pathological stage, molecular subtypes by IHC, and histological subtypes. For WGS results, we also corrected for log-transformed sequence coverage of tumor and normal samples (continuous). All interaction and multivariate tests (*P*_adj_) were done using the analysis of variance to compare the models with and without the extra term. Because continuous variables contain zeros, the logarithmic transformation was applied as follows: log_10_(*x* + 1). The Kruskal–Wallis test was used to test if *MYC* expression originates from the same population according to the status of *MYC*/*TP53* alterations. All correlations were calculated using the non-parametric Spearman’s *rho* coefficient. All reported *P* values were two-tailed. Multiple testing correction was performed using the false discovery rate method (FDR) [18], and differences were considered significant when the FDR was < 0.05. All analyses were done in R software version 3.3.3 (available at www.r-project.org) and Bioconductor version 3.6. Differential expression analysis was performed with DESeq2 v.1.14.1 R/Bioconductor package [19] on raw count data (20,724 genes). Significantly differentially expressed genes were selected with a FDR of < 0.1, independent filtering was performed using default parameters to select a set of genes for multiple test correction which maximizes the number of adjusted *P* values less than a given critical value alpha (by default 0.1) and differentially expressed genes were identified by using the default cutoff of *P* value adjusted for multitesting < 0.1. We used gage v.2.24.0 R/Bioconductor package [20] to identify significantly enriched pathways from the Kyoto Encyclopedia of Genes and Genomes (KEGG) [21] and biological process from Gene Ontology with the log2FoldChange from DEseq2 results as input data.

## Results

### Association between clinicopathological variables, parity, and age at first pregnancy

From a publicly available dataset comprising 560 breast cancer patients [14], a total of 313 with available information on parity were included. We identified 264 (84.3%) parous and 49 (15.7%) nulliparous patients (Additional file 2: Figure S1). In the parous group, 153 patients (57.9%) had available information on age at first pregnancy (median of 25 years, range 16–46 years). Parous patients were divided into two groups: 82 early and 71 late parous patients by using the median age at first pregnancy as a cutoff value. All patients had available somatic mutations and SCNAs data, 182 patients (58.1%) had available transcriptomic data, and 170 patients (54.3%) had information on TIL levels (Additional file 2: Figure S2).

Table 1 summarizes the clinicopathological features of patients. Compared to parous patients, nulliparous patients had larger tumors (tumor size > 2 cm, 59.2% vs. 37.1%, *P* = 0.006), higher frequency of lymph node positive disease (40.8% vs. 27.7%; *P* = 0.027), and lower frequency of triple negative breast cancer (TNBC) (4.1% vs. 23.2%; *P* = 0.001). Compared to early parous patients, late parous patients had a younger age at breast cancer diagnosis (median, 49 years; range, 28–81 years vs. median, 59 years; range, 34–81 years; *P* = 2.58 × 10^−5^) and were more often pre-menopausal (45.3% vs. 20.9%; *P* = 0.005). Late parous patients also had smaller tumors (tumor pathological size > 2 cm, 40.8% vs. 51.2%, *P* = 0.012), lower frequency of TNBC (19.7% vs. 39%, *P* = 0.026), and higher frequency of lobular histological subtype (14.5% vs. 2.5%, *P* = 0.01).

**Table 1:** Clinicopathological features of nulliparous and parous patients

*N*	Nulliparous	Parous	*P*	Early parous	Late parous	*P*
	49	264		82	71	
Age at diagnosis	54 (30–81)	55 (28–81)	0.796^a^	59 (34–81)	49 (28–81)	2.6 × 10^–5a^
Menopausal status						
Pre	13 (33.3%)	60 (30.3%)	0.85	14 (20.9%)	29 (45.3%)	0.0053
Post	26 (66.7%)	138 (69.7%)		53 (79.1%)	35 (54.7%)	
Stage						
I	7 (14.9%)	41 (15.9%)	0.019	6 (7.6%)	17 (24.3%)	0.039
II	20 (42.6%)	81 (31.4%)		33 (41.8%)	27 (38.6%)	
III	11 (23.4%)	30 (11.6%)		12 (15.2%)	10 (14.3%)	
IV	0 (0%)	1 (0.4%)		1 (1.3%)	0 (0%)	
Na	9 (19.1%)	105 (40.7%)		27 (34.2%)	16 (22.9%)	
pT						
Tx	9 (18.4%)	105 (39.8%)	0.0062	27 (32.9%)	16 (22.5%)	0.012
≤ 2 cm	11 (22.4%)	61 (23.1%)		13 (15.9%)	26 (36.6%)	
> 2 cm	29 (59.2%)	98 (37.1%)		42 (51.2%)	29 (40.8%)	
pN						
Nx	11 (22.4%)	112 (42.4%)	0.027	30 (36.6%)	17 (23.9%)	0.2
N0	18 (36.7%)	79 (29.9%)		25 (30.5%)	23 (32.4%)	
N1+	20 (40.8%)	73 (27.7%)		27 (32.9%)	31 (43.7%)	
Grade						
I	6 (14%)	29 (12.7%)	0.93	8 (9.8%)	5 (7%)	0.059
II	17 (39.5%)	86 (37.7%)		25 (30.5%)	35 (49.3%)	
III	20 (46.5%)	113 (49.6%)		49 (59.8%)	31 (43.7%)	
Subtype by IHC						
Lum A-like	22 (44.9%)	106 (40.3%)	0.0013	29 (35.4%)	39 (54.9%)	0.026
Lum B-like	19 (38.8%)	54 (20.5%)		20 (24.4%)	17 (23.9%)	
HER2+/HR+	6 (12.2%)	29 (11%)		0 (0%)	0 (0%)	
HER2+/HR-	0 (0%)	13 (4.9%)		1 (1.2%)	1 (1.4%)	
TNBC	2 (4.1%)	61 (23.2%)		32 (39%)	14 (19.7%)	
Histology						
Ductal	36 (76.6%)	203 (81.2%)	0.67	71 (88.8%)	49 (71%)	0.01
Lobular	5 (10.6%)	23 (9.2%)		2 (2.5%)	10 (14.5%)	
Other	6 (12.8%)	24 (9.6%)		7 (8.8%)	10 (14.5%)	

*pT* pathological tumor size, *pN* pathological nodal status, *HR* hormone receptor, *P P* value derived from *χ*^2^ test or the Fisher exact test when appropriate

^a^Except continuous variable derived from Mann–Whitney *U* test

In the following sections, we investigated the imprint of parity and age at first pregnancy on breast cancer biology by using a systematic multivariate analysis adjusted for potential confounders, namely age at diagnosis, pathological stage, molecular subtypes by IHC, and histological subtypes.

### The influence of parity and age at first pregnancy on the mutational landscape of breast cancer

**Fig. 1:**
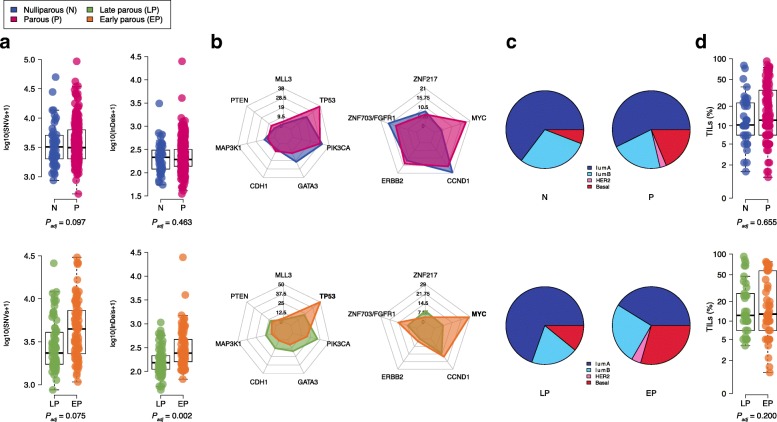
Imprint of pregnancy and age at first pregnancy on breast cancer biology. **a** Comparison of somatic SNVs and Indels in tumor between nulliparous (*n* = 49) and parous (upper) (*n* = 264) and between early (*n* = 82) and late parous (bottom) (*n* = 71). *P*_adj_, *P* values derived from multivariate linear regression analysis adjusted for potential confounders. **b** Radar plots showing the frequency of somatic driver mutations and somatic driver SNCAs in breast cancer from nulliparous (*n* = 49) and parous (upper) (*n* = 264) and between early (*n* = 82) and late parous (bottom) (*n* = 71) patients. Significant genes independently associated with parity or age at first pregnancy are highlighted in bold. **c** Proportion of PAM50 breast cancer subtypes in nulliparous (*n* = 34) and parous (upper) (*n* = 148) and in early (*n* = 51) and late parous (bottom) (*n* = 45) patients. **d** Comparison of TIL levels (%) between nulliparous (*n* = 26) and parous (upper) (*n* = 134) and between early (*n* = 47) and late parous (bottom) (*n* = 38) patients. *P*_adj_, *P* values derived from multivariate linear regression analysis adjusted for potential confounders

We first sought to investigate the imprint of parity and age at first pregnancy on somatic mutational load (Additional file 1: Table S1). There was no significant difference in the total number of substitutions (SNVs) according to parity nor age at first pregnancy (*P*_adj_ = 0.097, *P*_adj_ = 0.075, respectively, Fig. 1a). There was no significant difference in the total number of insertions or deletions (Indels) according to parity (*P*_adj_ = 0.464, Fig. 1a). In contrary, compared to tumors from late parous patients, tumors from early parous patients were significantly associated with a higher Indels load (*P*_adj_ = 0.002, FDR = 0.007, Fig. 1a). There was no significant difference between the total number of rearrangements according to parity nor age at first pregnancy (Additional file 1: Table S1).

We next interrogated the influence of parity and age at first pregnancy on the frequency of mutations in breast cancer driver genes. Among the driver mutated genes, seven had at least one non-silent mutation with a frequency of > 5% across the whole cohort (Additional file 1: Table S2). As expected, *PIK3CA* and *TP53* were the most frequently mutated genes (Fig. 1b). None of the driver mutated genes were associated with parity in the multivariate analysis. However, in the parous group, early age at first pregnancy was independently associated with higher frequency of *TP53* mutations (41/82 (50%) vs. 16/71 (22.5%); *P*_adj_ = 0.010; FDR = 0.046, Fig. 1b) and lower frequency of *CDH1* mutations (1/82 (1.2%) vs. 9/71 (12.7%); *P*_adj_ = 0.013; FDR = 0.046, Fig. 1b). Considering the distribution of *TP53* mutations type, early parous patients had a significantly higher frequency of truncating mutations as compared to late parous patients (21/82 (25.6%) vs. 5/71 (7%); *P*_adj_ = 0.014, Additional file 2 : Figure S3). Altogether, our results show that age at first pregnancy is associated with biological differences in the mutational landscape of subsequent breast tumors with early parity associated with higher Indels burden and higher frequency of deleterious mutations in *TP53* gene*.*

### The influence of parity and age at first pregnancy on somatic copy number alterations

**Fig. 2:**
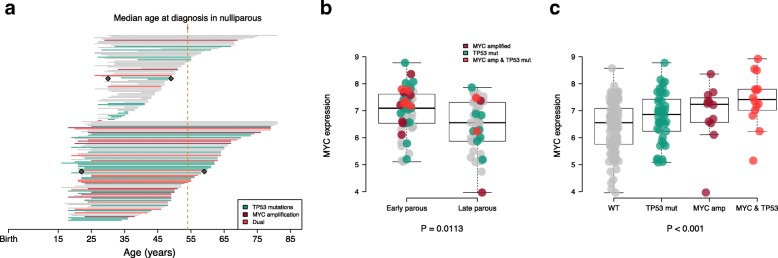
Co-occurrence of *MYC* amplification and *TP53* mutations is associated with age at first pregnancy. **a** Timeline of 153 patients with available data on age at first pregnancy. Each line represents an individual patient from age at first pregnancy (start of the line) to age at breast cancer diagnosis (end of the line). Late parous patients (upper) (*n* = 71) and early parous patients (bottom) (*n* = 82) are grouped according to median age at first pregnancy (25 years). Gray diamond represents the median age at first pregnancy and at diagnosis in the two groups. Lines are colored according to *TP53* mutations (green) *MYC* amplification (dark red) and the co-occurrence of both (red). **b** Comparison of *MYC* expression in early (*n* = 51) and late parous patients (*n* = 45). Each dot represents an individual patient and is colored according to *TP53* mutations (green) *MYC* amplification (dark red) and the co-occurrence of both (red). *P* value is derived from the multivariate linear regression analysis adjusted for potential confounders. **c** *MYC* expression according to *TP53* mutations, *MYC* amplification or the co-occurrence of both. *P* value is derived from the Kruskal–Wallis test

Somatic copy number alterations (SCNAs) play a major role in breast cancer biology [22, 23]. We identified five driver genes with a frequency of SCNAs > 5% across all patients (Additional file 1: Table S3). *MYC* tended to be more frequently amplified in parous than in nulliparous patients (49/264 (18.6%) vs. 2/49 (4.1%); *P*_adj_ = 0.052; FDR = 0.26, Fig. 1b). In the parous group, *MYC* amplification was significantly more frequent in the early parous group than in the late parous group (23/82 (28%) vs. 5/71 (7%); *P*_adj_ = 0.008; FDR = 0.040, Fig. 1b). When evaluating the co-occurrence of SCNAs and somatic mutations, we found that co-occurrence of *MYC* amplification and *TP53* mutations was independently associated with age at first pregnancy, with early parous patients having a higher frequency of simultaneous alterations of *MYC* and *TP53* genes (15/82 (18.3%) vs. 3/71 (4.2%); *P*_adj_ = 0.087, Fig. 2a and Additional file 2: Figure S3). Taken together, these results suggest that age at first pregnancy may also shape the somatic copy number alteration profiles of subsequent breast cancer.

### The influence of parity and age at first pregnancy on BRCAness

We investigated the BRCAness status of tumors according to reproductive history. Since germline BRCA1/2 mutation status was not available for all samples, we used HRDetect to identify *BRCA1*/*BRCA2*-deficient samples [15]. We did not find any significant differences in the proportion of *BRCA1*/*BRCA2*-deficient patients between nulliparous and parous groups (6/49 (12.2%) vs. 50/264 (18.9%), respectively, *P*_adj_ = 0.386) nor between early and late parous group (25/82 (30.5%) vs. 14/71 (19.7%), respectively, *P*_adj_ = 0.473). Thus, reproductive history and age at first pregnancy do not seem to affect homologous recombination DNA repair capacity in subsequent breast cancer.

### Integrative analysis of the genomic alterations and the transcriptomic profiles associated with parity and age at first pregnancy

RNA sequencing data were available for a subset of 182 patients, of which 34 were nulliparous (Additional file 2: Figure S2 and Additional file 1: Table S4). We first determined the intrinsic molecular subtype distribution of breast cancer using the PAM50 classifier [24]. We did not find a significant difference in the distribution of the PAM50 subtypes between nulliparous and parous (Fig. 1d). In contrast, early parous patients had a higher proportion of basal-like subtype tumors (15/51 (29.4%) vs. 4/45 (8.9%); *P* = 0.009, Fig. 1d, Additional file 1: Table S4). In order to identify de novo gene expression profiles that might be associated with parity and age at first pregnancy, we performed a multivariate differential expression analysis using DEseq2 [19] controlling for age at diagnosis, pathological stage, molecular subtypes by IHC, and histological subtypes. A total of 62 genes were differentially expressed between nulliparous and parous (Additional file 1: Table S5). Among these genes, three were associated with mammary development; *OXTR* and *ATP2B2* were downregulated whereas *NRG3* was upregulated in nulliparous. Pathway analysis using the generally applicable gene-set enrichment (GAGE) analysis [20] revealed an enrichment of genes related to extracellular matrix (ECM) receptor interaction in parous (Additional file 1: Table S6). When comparing early and late parous patients, 466 genes were differentially expressed, among which 305 were upregulated in early parous (Additional file 1: Table S7). However, pathway analysis did not reveal any significant enrichment of relevant biological processes (Additional file 1: Table S8).

Due to the higher frequency of *MYC* amplification in early parous patients, we determined if this would also impact *MYC* at the mRNA expression levels. Early parous patients were independently associated with an upregulation of *MYC* expression (*P*_adj_ = 0.0113, Fig. 2b). We also evaluated the expression of *MYC* according to *TP53* mutations and *MYC* amplification and found that *MYC* expression was the highest in tumors harboring concurrent *TP53* mutation and *MYC* amplification (Fig. 2c).

### The influence of parity and age at first pregnancy on tumor immune microenvironment

Previous reports have hypothesized that the pregnancy-induced tumor protection could be attributable to an improved anti-tumor immunity [25–29]. Therefore, we assessed whether reproductive history could be associated with tumor-infiltrating lymphocyte (TIL) levels that is considered as a surrogate of tumor immunogenicity (Additional file 1: Table S9). We did not find any significant difference in the proportion of stromal TILs according to parity or according to the age at first pregnancy (*P*_adj_ = 0.655; *P*_adj_ = 0.200, respectively, Fig. 2d). Similarly, no differences were observed when comparing intratumoral TILs according to parity or age at first pregnancy (*P*_adj_ = 0.240; *P*_adj_ = 0.889, Additional file 1: Table S9). Thus, reproductive history does not seem to influence breast tumor immune microenvironment.

### Pregnancy-associated breast cancer is associated with increased TIL infiltration

**Fig. 3:**
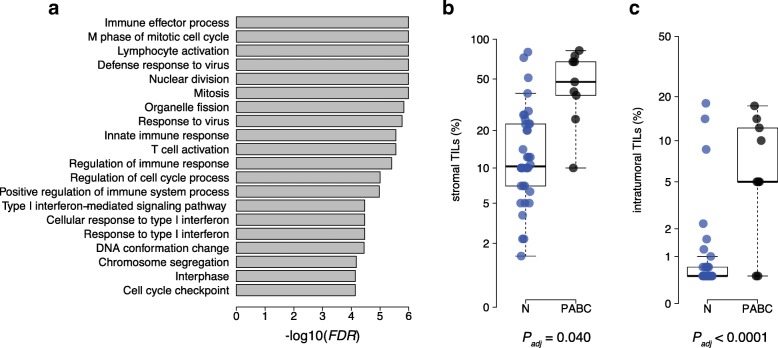
PABC patients are associated with higher TIL levels. **a** Results from the GAGE analysis showing the top 20 most significant biological processes enriched in PABC patients. **b** Comparison of stromal and **c** intratumoral (right) TIL levels (%) between nulliparous (*n* = 49) and PABC (*n* = 17). *P*_adj_, *P* values derived from multivariate linear regression analysis adjusted for potential confounders

Pregnancy-associated breast cancer (PABC) can be defined as cases diagnosed up to 10 years postpartum [30]. In this cohort, we identified 17 PABC patients and compared them with 49 nulliparous patients. Compared to nulliparous, PABC patients had a younger age at diagnosis (median, 38 years; range, 28–48 years vs. median, 54 years; range, 30–81 years; *P* = 5.79 × 10^−6^, Additional file 1: Table S10) and higher frequency of TNBC (5/17 (29.4%) vs. 2/49 (4.1%); *P* = 0.021, Additional file 1: Table S10). We did not find any significant differences between PABC and nulliparous in the pattern of somatic mutations or somatic copy number alterations (Additional file 1: Table S1-S3). Nine PABC had available gene expression and TIL scoring. At the transcriptomic level, we found that PABC patients were associated with enrichment of biological processes related to immune function (Fig. 3a and Additional file 1: Table S11). Moreover, PABC patients had an increased lymphocytic infiltration both for stromal and intratumoral TIL levels (*P*_adj_ = 0.040 and *P*_adj_ < 0.0001, respectively; Fig. 3b, c). Taken together, these results indicate that cancer occurring in the postpartum breast is associated with increased TIL levels.

## Discussion

To our knowledge, this is the first study that explores the impact of reproductive history on the genomic landscape and the immune composition of subsequent breast cancer. While previous studies documented the risk of developing breast cancer according to reproductive history [1–5], this analysis provides further insights on the differences at the pathologic, genomic, transcriptomic, and immunologic levels according to prior parity and age at first pregnancy. Independently of clinicopathological features, our findings indicate that age at first pregnancy impacts the genomic makeup of subsequent breast cancer. Early parous patients developed tumors characterized by a higher number of Indels, a lower frequency of *CDH1* mutations, a higher frequency of *TP53* mutations and *MYC* amplification, while PABC patients exhibited higher TIL infiltration.

The higher proportion of TNBC in parous and particularly in early parous patients could be attributed to a differential effect of pregnancy-induced tumor protection according to breast cancer subtypes. We and others have documented that the pregnancy-induced tumor protection is different according to breast cancer subtypes with parity and young age at first pregnancy being associated with a marked reduction in the risk of developing luminal subtype [7–10].

Our study reveals that age at first pregnancy has a bigger imprint on genomic alterations of breast cancer than parity status alone. The apparent lack of impact of parity *per se* could be due to the relatively low number of nulliparous patients. An alternative explanation could be that breast cancer from late parous resembles breast cancer from nulliparous women. Therefore, the lack of difference between nulliparous and parous could be related to the fact that the parous group encompasses late parous patients resulting in a possible dilution of the signal.

**Fig. 4:**
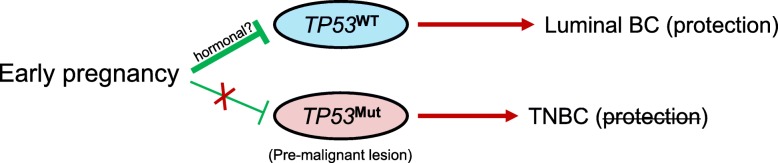
Proposed model explaining the difference of parity-induced protection according to breast cancer subtypes. *TP53* has long been recognized as a potential mediator of pregnancy-induced resistance to mammary carcinogenesis. We hypothesize that an early pregnancy might protect less effectively against *TP53* mutant premalignant lesion. *TP53* mutations are highly linked to the TNBC subtype; this could explain why the pregnancy-induced resistance is lost in TNBC

At the gene level, early parous patients had a higher frequency of *TP53* mutation, *MYC* amplification, and a lower frequency of *CDH1*. Interestingly, the co-occurrence of *TP53* mutations and *MYC* amplification was independently associated with age at first pregnancy, while the proportion of truncating *TP53* mutations was higher in early parous patients. We observed that tumors harboring concurrent *MYC* amplification and *TP53* mutation had the highest *MYC* expression. This observation is in line with a recent investigation of the *MYC* oncogene in pan-cancer data [31]. Previous reports have suggested that *TP53* mutations are a common mechanism that disturbs the apoptotic pathway in *MYC*-driven tumors [32]. It has been hypothesized that overexpression of *MYC* induces *TP53*-dependent apoptosis, and, as a consequence, *MYC*-driven tumors often require dysregulation of the apoptotic pathway to promote proliferation [33]. *TP53* has long been recognized as a potential mediator of pregnancy-induced resistance to mammary carcinogenesis. It has been shown in mice that p53 and its downstream transcriptional target p21 are increased in parous and estrogen/progesterone-treated mammary epithelium in response to a carcinogen [34]. In the absence of p53, the protection given by parity or exogenous hormones is lost [35–37]. We hypothesize that the higher frequency of *TP53*-mutant breast cancer observed in early parous women could be explained by the fact that an early pregnancy could protect less effectively against *TP53* mutant pre-malignant lesions (Fig. 4). In breast cancer, *TP53* mutations are highly linked to molecular subtype with a frequency of 80% in basal-like compared to 26% in luminal tumors [23]. The lower susceptibility of *TP53*-mutant premalignant lesions to the protective effect of an early parity might be the underlying cause of the known differential effect of parity-induced protection according to tumor subtypes.

*CDH1* mutations have been associated with invasive lobular breast cancer subtype [38]. As the multivariate analysis was adjusted for histological subtypes, the lower frequency of *CDH1* mutations observed in early parous patients cannot be explained by differences in histological subtypes. Finally, in line with prior studies [39, 40], our data do not show an impact of parity and age at first birth on BRCA-related breast cancer risk.

Another hypothesis underlying the protection associated with an early pregnancy argues that the high level of circulating hormones associated with pregnancy would induce differentiation of the mammary gland while decreasing the tumorigenic potential of breast cells [41]. Gene expression studies comparing nulliparous and parous normal mammary tissue, from both rat and human, have observed upregulation of genes related to cell differentiation in the parous breast [42, 43]. According to this theory, a full-term pregnancy early in reproductive life would induce a molecular switch in mammary stem cells leading to a permanent decrease in their proliferation potential and resistance to oncogenic transformation [11, 44]. The results from our bulk RNA-seq analysis of tumor samples are not in line with this hypothesis, but we believe that future studies using single-cell RNA-seq would improve our understanding of the mechanism of the parity-induced protection against breast cancer. Noteworthy, we found that the expression of *OXTR* was higher in parous compared to nulliparous patients. In the normal tissue of parous women, the gene encoding oxytocin receptor (*OXTR*) is physiologically upregulated during lactation and has been shown to remain overexpressed later in life [12, 45]. However, due to the lack of functional studies, it is not clear whether this gene is involved in breast cancer tumorigenesis or simply related to physiological changes induced by pregnancy. The enrichment of genes related to ECM receptor interaction in parous patients might be related to involution, a profound physiological change in the mammary gland after pregnancy. Right after breastfeeding the fully differentiated gland regresses to its pre-pregnant state by an innate tissue-remodeling mechanism. Evidence indicates that involution is mediated in part by ECM-degrading proteinases, leading to basement membrane degradation and subsequent apoptosis of the unwanted secretory epithelial cells [46, 47]. The exact role of the enrichment of genes related to ECM in parous patients on human breast cancer biology has still to be determined, but involution, that shares similarities with inflammation and wound healing programs, has been shown to promote breast cancer progression and metastasis in several animal models [46, 48].

Finally, previous reports have hypothesized that the pregnancy-induced tumor protection could be attributable to an improved anti-tumor immunity [25–29]. Our analysis reveals no differences in TILs infiltration levels according to parity or age at first pregnancy. The existence of a more complex immune component related to reproductive history cannot be excluded, but it is not supported by our analysis. Previous reports have documented that the immune milieu of the postpartum mammary gland associated with involution could promote tumorigenesis [49–51]. We observed an increase of TILs levels in PABC patients but the composition of the immune infiltrate has still to be determined to validate this hypothesis.

A potential limitation of our study is the lack of data on other reproductive factors (e.g., breastfeeding, age at menarche, and time since last pregnancy) that could also potentially imprint the biology of breast cancer. Indeed, breastfeeding and age at menarche have been linked to breast cancer risk but since they are often self-reported, they are more difficult to assess reliably [7]. The lack of validation using an independent cohort is another important limitation. We retrieved the clinical information from the three independent cohorts of breast cancer with publicly available genomic data [23, 52, 53], but unfortunately, reproductive variables were missing, which precluded the validation of our findings. Another limitation is the absence of HER2+ subtype in the transcriptomic analysis.

## Conclusions

In conclusion, our findings highlight an unprecedented link between reproductive factors and the genomic landscape of subsequent breast cancer. We further hypothesize that *TP53*-mutant premalignant lesions could be less susceptible to the protective effect of an early parity, which might explain the difference of parity-induced protection according to breast cancer subtypes. Our results that need to be validated in other studies support that patients’ reproductive history should be routinely collected in future large-scale genomic studies addressing the biology of female cancers.

## Additional files

Additional file 1: **Table S1.** Systematic multivariate analysis of mutational load comparing nulliparous vs. parous, early parous vs. late parous and PABC vs. nulliparous patients. **Table S2.** Systematic multivariate analysis of breast cancer driver SNVs comparing nulliparous vs. parous, early parous vs. late parous and PABC vs. nulliparous patients. **Table S3.** Systematic multivariate analysis of breast cancer driver SCNAs comparing nulliparous vs. parous, early parous vs. late parous and PABC vs. nulliparous patients. **Table S4.** Clinicopathological features of nulliparous and parous patients included in the RNAseq analysis. **Table S5.** Differential expression analysis between nulliparous and parous patients using DEseq2 on raw counts data and controlling for age at diagnosis, pathological stage, molecular subtypes by IHC, histological subtypes. **Table S6.** Pathway analysis using the generally applicable gene-set enrichment (GAGE) method to identify significantly enriched pathways between nulliparous and parous patients. **Table S7.** Differential expression analysis between early and late parous patients using DEseq2 on raw counts data and controlling for age at diagnosis, pathological stage, molecular subtypes by IHC, histological subtypes. **Table S8.** Pathway analysis using the generally applicable gene-set enrichment (GAGE) method to identify significantly enriched pathways between early and late parous patients. **Table S9.** Systematic multivariate analysis of TILs levels comparing nulliparous vs. parous, early parous vs. late parous and PABC vs. nulliparous patients. **Table S10.** Clinicopathological features of nulliparous and PABC patients. **Table S11.** Pathway analysis using the generally applicable gene-set enrichment (GAGE) method to identify significantly enriched pathways between nulliparous and PABC patients. (XLSX 366 kb)

Additional file 2: **Figure S1.** Flowchart summarizing the number of patients included in the analyses and the reasons for inclusion and exclusion. **Figure S2.** Venn diagram summarizing the number of patients with available data. Figure S3. Genomic landscape of breast cancer according to pregnancy and age at first pregnancy. (PDF 239 kb)

## Abbreviations

TILs: Tumor-infiltrating lymphocytes
PABC: Pregnancy-associated breast cancer
SCNAs: Somatic copy number alterations
TNBC: Triple negative breast cancer
IHC: Immunohistochemistry
SNVs: Single-nucleotide variant
pT: Pathological tumor size
pN: Pathological nodal status
HR: Hormone receptor
*P*: *P* value
FDR: False discovery rate
