# Supplementary material for: Imprint of parity and age at first pregnancy on the genomic landscape of subsequent breast cancer
Source: Breast Cancer Res. 2019 Feb 15;21:25. doi: 10.1186/s13058-019-1111-6 (PMC6377756; doi:10.1186/s13058-019-1111-6)
Supplement: Supplementary file 2 — Figure S1. Flowchart summarizing the number of patients included in the analyses and the reasons for inclusion and exclusion. Figure S2. Venn diagram summarizing the number of patients with available data. Figure S3. Genomic landscape of breast cancer according to pregnancy and age at first pregnancy. (PDF 239 kb) [file 13058_2019_1111_MOESM2_ESM.pdf]

Imprint of pregnancy and age at first pregnancy on the genomic landscape of breast cancer

Additional file 2: Figure S1-S3

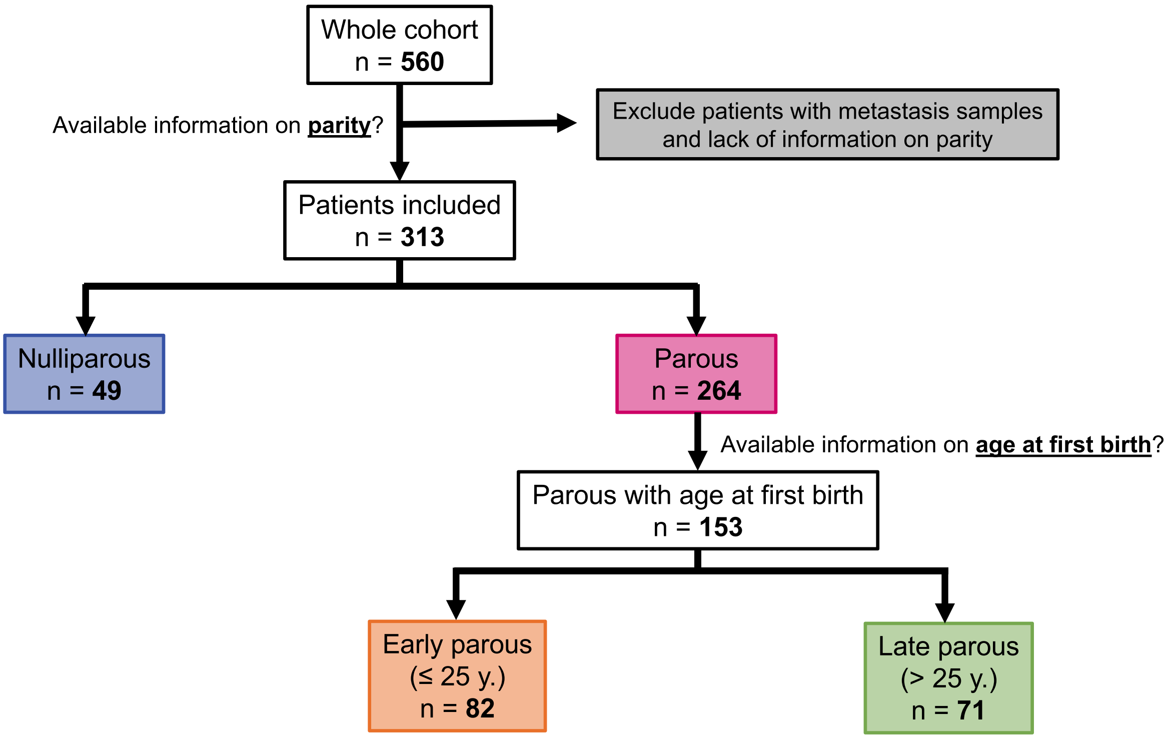

Figure S1. Flowchart summarizing the number of patients included in the analyses and the reasons for inclusion and exclusion

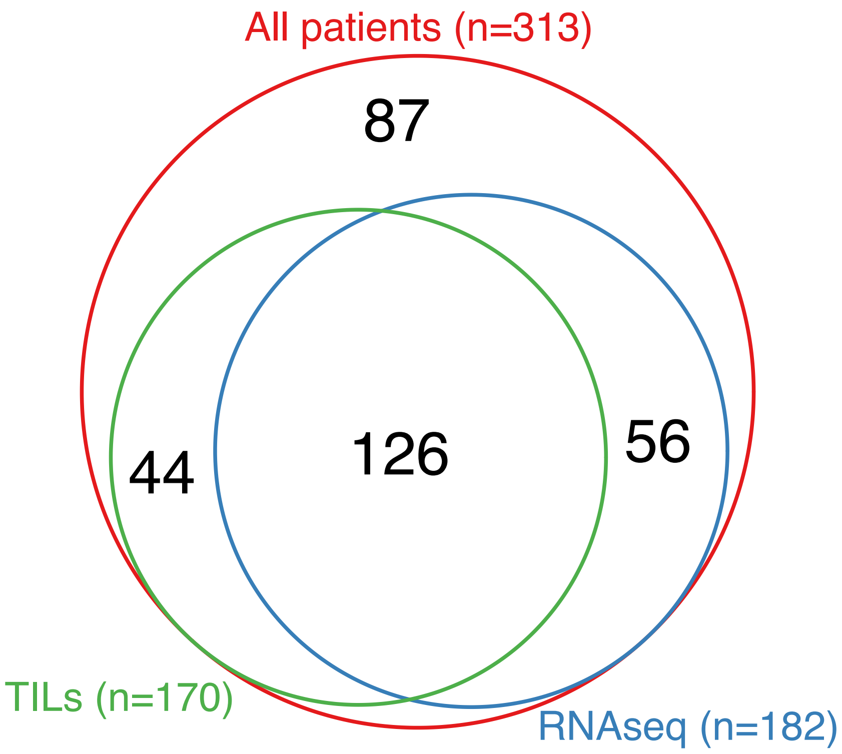

Figure S2. Venn diagram summarizing the number of patients with available data
313 patients (100%) had available somatic mutations and somatic copy number alterations (SCNAs) data (red circle) while 182 patients (58.1%) had available transcriptomic data (blue circle) and 170 patients (54.3%) had available information on TILs (green circle).

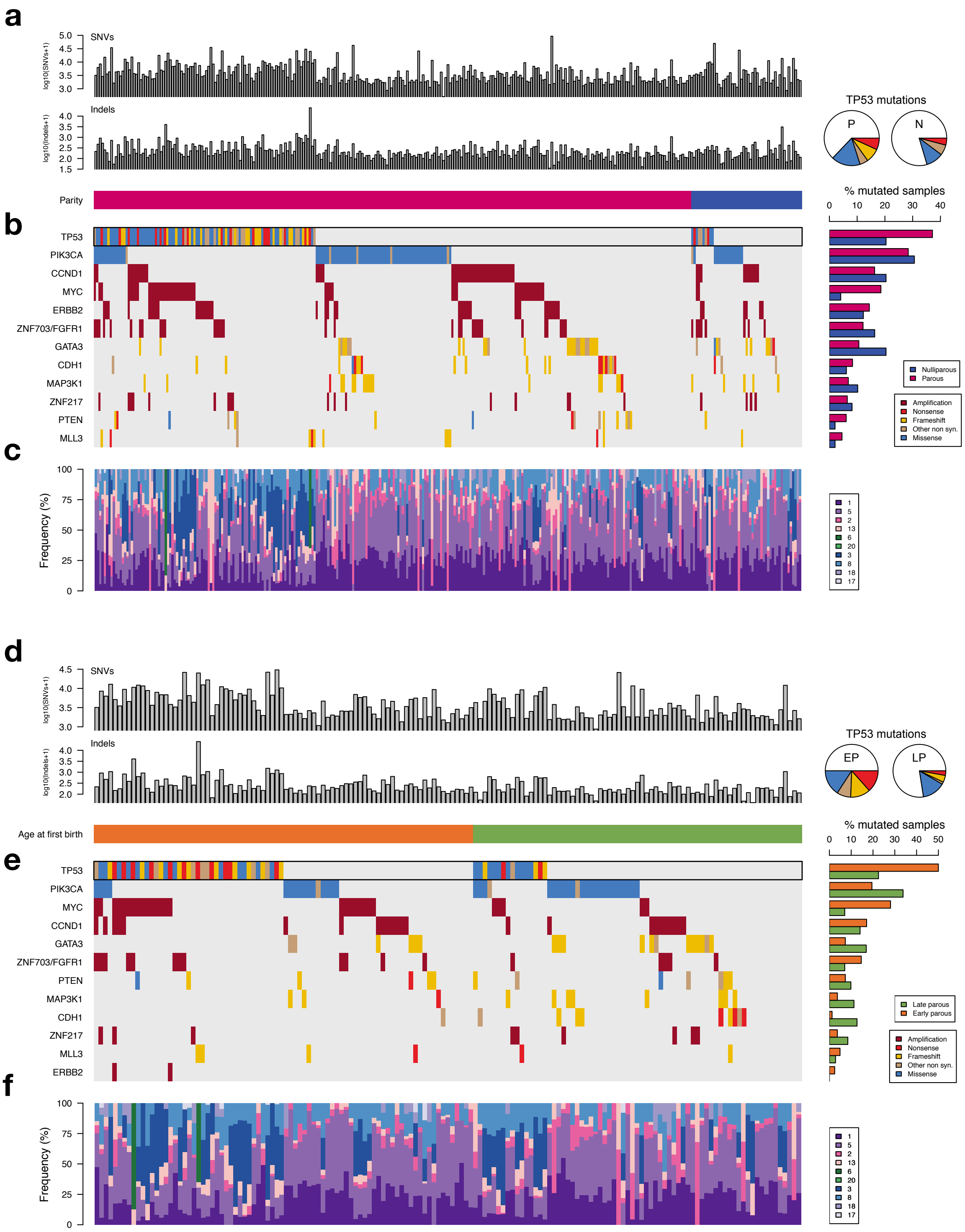
a
SNVs
log10(SNVs+1)
Indels
log10(Indels+1)
TP53 mutations
P
N
Parity
% mutated samples
b
TP53
PIK3CA
CCND1
MYC
ERBB2
ZNF703/FGFR1
GATA3
CDH1
MAP3K1
ZNF217
PTEN
MLL3
Nulliparous
Parous
Amplification
Nonsense
Frameshift
Other non syn.
Missense
c
Frequency (%)
d
SNVs
Indels
TP53 mutations
EP
LP
Age at first birth
% mutated samples
e
TP53
PIK3CA
MYC
CCND1
GATA3
ZNF703/FGFR1
PTEN
MAP3K1
CDH1
ZNF217
MLL3
ERBB2
Late parous
Early parous
Amplification
Nonsense
Frameshift
Other non syn.
Missense
f
Frequency (%)

Figure S3. Genomic landscape of breast cancer according to pregnancy and age at first pregnancy
(a) Bar chart representing the absolute number of substitutions and indels in nulliparous and parous patients.
(b) Co-mutation plot showing genes harboring at least one non-silent mutation with a frequency of at least 5% across the whole cohort, and their corresponding frequencies in nulliparous and parous patients.
(c) Proportion of breast cancer substitution signatures in each sample. Signatures are colored according to broad biological groups: 1 and 5 are associated with clock-like processes, 2 and 13 are APOBEC-related, 20 and 26 are associated with mismatch- repair deficiency, 3 and 8 are associated with homologous-recombination deficiency.
(d) Bar chart representing the absolute number of substitutions and indels in early and late parous patients.
(e) Co-mutation plot showing genes harboring at least one non- silent mutation with a frequency of at least 5% across the whole cohort, and their corresponding frequencies in early and late parous patients.
(f) Proportion of breast cancer substitution signatures in each sample. Signatures are colored according to broad biological groups: 1 and 5 are associated with clock-like processes, 2 and 13 are APOBEC-related, 20 and 26 are associated with mismatch- repair deficiency, 3 and 8 are associated with homologous-recombination deficiency.
